# Sentinel node‐assisted neck dissection in advanced oral squamous cell carcinoma—A new protocol for staging and treatment

**DOI:** 10.1002/cam4.5966

**Published:** 2023-04-21

**Authors:** Rusana Bark, Aeneas Kolev, Alexandra Elliot, Krzysztof Piersiala, Anders Näsman, Per Grybäck, Susanna Kumlien Georén, Malin Wendt, Lars Olaf Cardell, Gregori Margolin, Linda Marklund

**Affiliations:** ^1^ Department of Clinical Sciences Intervention and Technology, Division of ENT Diseases Karolinska Institute Stockholm Sweden; ^2^ Medical Unit Head Neck Lung and Skin cancer, Department of Head and Neck Surgery Karolinska University Hospital Stockholm Sweden; ^3^ Department of Otorhinolaryngology Karolinska University Hospital Stockholm Sweden; ^4^ Department of Oncology‐Pathology Karolinska Institutet Stockholm Sweden; ^5^ Department of Clinical Pathology Karolinska University Hospital Stockholm Sweden; ^6^ Department of Molecular Medicine and Surgery Karolinska Institutet Stockholm Sweden; ^7^ Department of Medical Radiation Physics and Nuclear Medicine Karolinska University Hospital Stockholm Sweden; ^8^ Department of Surgical Sciences, Section of Otolaryngology and Head and Neck Surgery Uppsala University Uppsala Sweden

**Keywords:** head and neck cancer, metastases, neck dissection, oral squamous cell carcinoma, sentinel node biopsy

## Abstract

**Background:**

Sentinel lymph node biopsy (SLNB) is used to improve the staging of and guide treatment in patients with early‐stage T1–T2 N0 oral squamous cell carcinoma (OSCC). The role of sentinel nodes (SNs) and the use of SN‐technique in advanced OSCC (T3–T4 and/or N+) remain to be evaluated. This study investigates the nodal drainage and the rate of positive SNs (SNs+) in all stages of OSCC.

**Materials and Methods:**

In total, 85 patients with T1–T4 OSCC diagnosed 2019–2021 were included. We used a prolonged interval between peritumoral injection of radionuclide and SPECT–CT to include all SNs.

**Results:**

Patients with advanced OSCC presented a higher proportion of contralateral lymphatic drainage and a higher rate of SN+ compared to patients with early‐stage disease. T3–T4 and N+ tumors presented a tendency for a higher rate of contralateral lymphatic drainage compared to T1–T2 and N0 tumors (*p* = 0.1). The prevalence of positive nodes (SNs+) was higher among patients with advanced disease, T3–T4 versus T1–T2 (*p* = 0.0398).

**Conclusion:**

SN‐assisted ND enables identification and removal of all SNs + and has the potential for more accurate staging and could possibly give prognostic advantages regarding regional recurrence for all OSCC patients, especially among those with advanced disease. The precise localization of the SNs + also suggests that a more individualized ND approach might be possible in the future even for patients with advanced OSCC.

## 1 INTRODUCTION

Oral squamous cell carcinoma (OSCC) is the most common head and neck cancer and accounts for approximately 380,000 new cases worldwide annually.^1^ OSCC has an increasing incidence, and in contrast to the most head and neck cancers, that incidence is increasing among younger adults without a history of tobacco or alcohol abuse.^2^, ^3^, ^4^, ^5^, ^6^ Despite advances in surgical and oncological treatment the prognosis for OSCC is still relatively unfavorable with a 5‐year relative survival of 68%,^7^ where younger patients seem to have a worse outcome compared to their older counterparts.^8^

Neck metastases (N+) is a strong negative prognostic factor in OSCC.^9^, ^10^, ^11^ For patients diagnosed with advanced OSCC, T3–T4 and N+, therapeutic ipsi‐lateral neck dissection (TND) is an accepted standard of treatment often followed by radio/chemotherapy.^12^ In the event of a midline tumor or tumor crossing the midline, bilateral selective neck dissection is often performed. In patients with early‐stage OSCC (T1–T2N0), occult metastases in the neck are present in up to 20%–40% of patients, therefore leading to elective neck dissection (END) as the standard of care for early‐stage OSCC in many medical centers. ^10^, ^13^, ^14^, ^15^, ^16^

After END and TND, the rate of recurrence in the neck is relatively high for patients with OSCC, 13%–30% in early‐stage and 40% in N+ disease. Combined therapy (post‐operative radiotherapy (RT) or chemo‐radiotherapy [CRT]) is used as a common treatment standard for OSCC stage II and above. A large proportion of the recurrences are found in the ipsilateral treated neck.^15^, ^17^, ^18^ The high proportion of nodal recurrence may indicate that the conventional END and TND have not included all lymph nodes in the neck levels or did not include the neck level with the presence of occult metastases, thus leaving residual occult metastases in the neck field. Also, a proportion of the nodal recurrences occur on the contralateral side, a part of the neck that is not covered during traditional END/TND or post‐operative RT or CRT.

In the last decade, sentinel lymph node biopsy (SLNB) has been introduced in OSCC as a less invasive method for the detection of occult metastases in patients with early‐stage OSCC (T1–T2 N0). SLNB including pre‐operative single‐photon emission computed tomography (SPECT–CT) also allows a good assessment of the patient's individual lymphatic drainage pattern, that is identification of the tumor draining lymph nodes (TDLNs) called sentinel nodes (SNs).^19^ For early‐stage OSCC, SLNB have a reported sensitivity of 75%–87% and a negative predictive value of 94%–98% in detecting occult metastasis.^19^, ^20^, ^21^ Thus, SLNB is considered a reliable and safe method to identify occult metastases in the neck for early‐stage T1–T2N0 OSCC.^21^, ^22^ Survival outcome for early‐stage OSCC seems to be comparable between SNLB and END, according to recent metanalysis,^22^ although results from ongoing randomized trials, such as NRG‐HN006, are pending.

Assessment of the lymphatic drainage from early‐stage OSCC illustrate contralateral lymph drainage in 12%–22% of patients with well lateralized tumors and as high as 71% in patients with midline tumors. ^19^, ^23^, ^24^ While the rate of occult contralateral cervical metastases is low for patients with early‐stage OSCC (3%–6%)^9^, ^18^, ^19^, ^21^, ^25^ it is relatively high in advanced T3–T4 and N+ disease (30%).^9^ Therefore, SNLB is suggested to allow better control of the occult contralateral metastases than END in early stage OSCC.^19^, ^25^

There is data showing that the tumor size and depth of invasion (DOI) correlates to the rate of occult metastases. The rate of the occult metastases in SLNB is higher in T2 versus T1 disease (37.8% vs. 18.5%),^19^ and DOI is associated with a higher rate of occult contralateral nodal metastasis.^19^, ^25^ This data indicates a plausible value of adding the SN‐technique for T3, T4 and N+ OSCC.

In this study, we have mapped lymphatic drainage and investigated the prevalence of occult metastases in SNs in patients with more advanced OSCC (T3–T4, N+) and compared to early stage T1–T2 N0 OSCC in a Stockholm‐Gotland (Sweden) cohort. Also, a technique with a prolonged interval between peritumoral injection of radionuclide and SPECT–CT was used to include all SNs in the neck, and in cases of advanced OSCC the neck field was rechecked with both gamma probe and ICG‐camera at the end of surgery to ensure no SNs were left behind. The aim of this study was to evaluate if SN‐assisted ND can be a step toward more accurate staging and in the future a more individualized treatment achieving better locoregional control for patients with advanced T3, T4 and N+ OSCC.

## 2 MATERIALS AND METHODS

### 2.1 Patient characteristics

Eighty‐five patients diagnosed with OSCC, both T1–T4 N0 and N+ disease, diagnosed at Karolinska University Hospital, Sweden, between 2019 and 2021 and surgically treated with intention to cure were included in the study.

At Karolinska University Hospital, SNLB is currently part of the standard oncological practice for staging and treatment of OSCC. Among early stage (T1–T2 N0) OSCC patients, SNLB was performed in all patients, and in case of a positive SN, a subsequent (completion) ND including levels 1–4/5 was performed on the neck were the SNLB was positive. Patients with advanced OSCC (clinically staged T3, T4 and N+ disease) underwent SN‐assisted neck dissection level 1–4 or 1–5 on the ipsilateral side and SNLB in the contralateral neck in case of contralateral drainage by SPECT–CT, followed by a contralateral ND in case of positive contralateral SN in a second‐stage surgery. In the case of a midline tumors or tumors crossing the midline, selective SN‐assisted ND was performed bilaterally on patients with N+ disease. In all patients, tumor localization and nodal status were staged by clinical examination/biopsy, computed tomography (CT) and/or magnetic resonance imaging (MRI) and fine needle aspiration cytology when needed. The staging, following the eighth TNM staging classification, was confirmed in a multidisciplinary conference before surgery. Based on the pathological results after surgery, the need for further adjuvant treatment (RT or CRT) was decided in a second multidisciplinary conference.

The sentinel node‐assisted ND was preceded by preoperative submucosal peritumoral injections (4–6 injections depending on the tumor's size and location) with a Tc‐99 m (technetium‐99) labeled tracer. Tilmanocept (Lymphoseek, Cardinal Health) was used until February 2021, and after that replaced by Nanocolloid (Nanocoll, GE Healthcare). Imaging of the head–neck and thorax with a strictly standardized SPECT–CT (single‐photon computed tomography with CT) was performed earliest 1 h after peritumoral injections.^26^ Surgery was performed up to 24 h after Tc‐99 m‐labeled tracer injection and the dose was adjusted in relation to the time to surgery. At the beginning of the surgery, fluorescent indocyanine green (ICG) dye (Verdye, Diagnostic Green GmbH) was injected peritumorally in the same way as Tilmanocept/Nanocolloid. Localization of SN was confirmed intraoperatively by gamma probe (EuroProbe, Euromedical Instruments) in combination with optical fluorescence detection by an integrated illuminator and HD camera (VITOM NIR/ICG, Stortz or Stryker SPY‐PHI). An ipsilateral modified neck dissection level 1–3 was performed with addition of further levels and/or lymph node biopsies depending on the location of SNs for patients with cN0 OSCC. Patients with N+ OSCC were treated with modified radical neck dissection (level 1–5). SNs within the neck field were excised separately, or in a few cases, marked with a suture in the neck specimen. At the end of the neck surgery, when the neck specimen was excised, the neck field was rechecked with both gamma probe and ICG‐camera to ensure no SNs were left behind. SNs on the contralateral side was identified and excised as SNLB. SNs were sent separately for pathological analysis according to the hospital SN‐protocol.^26^ In N+ OSCC the SN was regarded positive when a SN other than the known metastasis presented macro/micro or isolated tumor cells in the histopathological report of the ND.

### 2.2 Statistical analysis

Statistical analysis was performed with the GraphPad Software, version 9.0.0. The Fisher's exact test was used to test the dependence between clinicopathological descriptive features and lymphatic drainage pattern and SN positivity. *p*‐value <0.05 was considered significant.

## 3 RESULTS

The study included a total of 85 patients with OSCC treated with SN‐assisted surgery. Overall, 63 patients were diagnosed with early‐stage T1–T2N0, and 22 patients were staged with advanced OSCC, T3–T4 and/or N+. Out of all 85 patients, 71 patients were staged preoperatively with N0 and 14 patients with N+ disease. None of the patients had contralateral neck metastases (N2c) or had distant metastases at the time of diagnosis. Patient characteristics are shown in Table 1. As expected, patients with early‐stage (T1–T2) OSCC were significantly more often diagnosed with N0 disease, as compared to patients with T3–T4 (63/70 (90%) versus 8/15 (53%), *p* = 0.002, 95% CI 2.126–25.66). In all 14 patients with N+ OSCC, the known neck metastases were identified with SPECT–CT before the surgery, as well as with both gamma probe and by ICG during surgery, and later verified in the histopathology report.

**TABLE 1:** Patient and tumor characteristics.

Patients and tumor characteristics												
	Lymphatic drainage according to SPECT CT						SN according to histopathology					
	All patients		Ipsilateral only		Bilateral		All patients		SN‐		SN+	
	*n*	%	*n*	%	*n*	%	*n*	%	*n*	%	*n*	%
Number of patients	85		58		27		84		54		30	
Age mean (range)	62 (22–90)		69		67		62 (22–90)		59		66	
Sex												
Female	37	43.5	24	41.4	13	48.1	36	42.9	26	48.1	10	33.3
Male	48	56.5	34	58.6	14	51.9	48	57.1	28	51.9	20	66.7
T												
T1	40	47.1	29	50.0	11	40.7	39	46.4	29	53.7	10	33.3
T2	30	35.3	21	36.2	9	33.3	30	35.7	19	35.2	11	36.7
T3	11	12.9	6	10.3	5	18.5	11	13	5	6.3	6	20.0
T4	4	4.7	2	3.5	2	7.4	4	4.8	1	1.9	3	10.0
**N**												
N0	71	83.5	50	86.2	21	77.8	71	84.5	48	88.9	23	76.7
N+	14	16.5	8	13.8	6	22.2	13	15.5	6	11.1	7	23.3
N1	6	7.1	4	6.9	2	7.4	5	6.0	3	5.6	2	6.7
N2b	8	9.4	4	6.9	4	14.8	8	9.5	3	5.6	5	16.7
Site of primary tumor												
Oral tongue (C021‐C023)	62	72.9	44	75.9	18	66.7	61	72.6	41	75.9	20	66.7
Floor of mouth (C040‐C049)	8	9.4	2	3.5	6	22.2	8	9.5	4	7.4	4	13.3
Lower alveolus and gingiva (C031)	6	7.1	5	8.6	1	3.7	6	7.1	4	7.4	2	6.7
Buccal mucosa (C060‐C061)	6	7.1	6	10.3	0	0	6	7.1	4	7.4	2	6.7
Others (C030, C044, C051)	3	3.5	1	1.7	2	7.4	3	3.6	1	1.9	2	6.7
Tillmanocept (T)/Nanocoll (Na)												
T	46	54.1	30	51.7	16	59.3	46	54.8	27	50.0	19	63.3
Na	39	45.9	28	48.3	11	40.7	38	45.2	27	50.0	11	36.7

### 3.1 Lymphatic drainage in OSCC

The lymphatic drainage seen by SPECT–CT was analyzed. Of the 85 patients, 58/85 (68%) presented with ipsilateral drainage only, while 27/85 (32%) also demonstrated a contralateral drainage. No patient had lymphatic drainage solely to the contralateral side of the neck (Table 1). Patients with early‐stage OSCC (T1–T2N0) showed a trend towards less contralateral tumor drainage, as compared to patients with advanced stages (N+, T3–T4) (*p* = 0.1).

### 3.2 Lymphatic drainage in relation to diagnosis

Patients diagnosed with the tumors in buccal mucosa and mandibular gingiva (C060, C031, *N* = 12), showed a trend (*p* = 0.09) towards less contralateral drainage compared to patients with tumors in the oral tongue and the floor of the mouth (C020‐23, C040‐49, *N* = 70) (Tables 1, 2).

**TABLE 2:** Comparison of T‐stage, location of the tumor and lymphatic drainage according to SPECT–CT in oral cancer patients with (a) clinically N0 disease; (b) clinically N0 and N+ disease.

		Ipsilateral drainage only, *n* (%)	Contralateral drainage, *n* (%)	*p*‐value^a^
a. Patients with clinically N0 disease				
T‐stage				
T1+T2		46 (73%)	17 (27%)	0.2243
T3+T4		4 (50%)	4 (50%)	
Location of tumor				
Oral tongue and floor of the mouth		43 (69.4%)	19 (30.6%)	0.451
The buccal and mandibular gingival tumors		7 (77.8%)	2 (22.2%)	
b. All patients (both clinically N0 and N+ disease)				
T‐stage				
T1+T2		50 (71.4%)	20 (28.6%)	0.2233
T3+T4		8 (53.3%)	7 (46.7%)	
Location of tumor				
Oral tongue and floor of the mouth		47 (65.3%)	25 (34.7%)	0.2105
The buccal and mandibular gingival tumors		11 (84.6%)	2 (15.4%)	

^a^ Fisher's exact test.

### 3.3 Lymphatic drainage in relation to T‐stage

Smaller tumors (T1–T2) tended to present more often with only ipsilateral lymphatic drainage compared to larger tumors (T3–T4), regardless of N0/N+ stage, (*p* = 0.2) (Table 2). In fact, the prevalence of contralateral lymph drainage increased in parallel to an escalation in T‐stage (Table 3).

**TABLE 3:** Contralateral lymph drainage according to SPECT–CT in relation to T and N stage.

T‐stage	Total	N0	N+
ALL	85	21/71 (29.6%)	6/14 (42.9%)
T1	11/40 (27.5%)	9/37 (23.7%)	1/3 (33.3%)
T2	9/30 (30%)	8/26 (30.8%)	2/4 (50%)
T3	5/11 (45.5%)	3/7 (42.9%)	2/4 (50%)
T4	2/4 (50%)	1/1 (100%)	1/3 (33.3%)

Among T1 and T2 patients with contralateral drainage, 11/11 (100%) of the T1 cases and 8/9 (89%) of the T2 cases had tumors anatomically located close to the midline or diagnosed with tumors in the oral tongue or floor of the mouth (C021, C040). In patients with larger tumors (T3–T4), 7/15 (47%) had lymphatic drainage also seen in the contralateral side (Table 3). Six out of seven T3–T4 OSCCs were diagnosed with tumors located in the oral tongue or floor of mouth (C021, C040), two of them exceeding the midline and three of them 3/6 (50%) were anatomically located close to the midline according to the patients' charts.

### 3.4 Lymphatic drainage in relation to N‐stage

None of the 14 patients with N+ OSCC at the time of diagnosis presented with metastases/N+ in the contralateral neck in the preoperative investigation. In all patients with N+ OSCC, the known metastases were identified as SN in the preoperative SPECT–CT and confirmed with both gamma probe and ICG during ND‐surgery (Tables 1, 2 and 3).

The rate of contralateral lymph drainage was higher in patients with N+ compared to N0 OSCC. Among all patients with N0 OSCC, 21/71 (29.6%) presented with contralateral drainage compared to 6/14 (42.9%) of all patients with N+ disease (Table 2). The rate of contralateral drainage was not affected by the T‐stage for patients with N+ disease since the contralateral drainage was the same for both T1–T2 N+ and for T3–T4 N+ (3/7 [42.8%]). Among the patients with N1 disease, contralateral lymph drainage was present in 2/6 (33%) patients compared to 4/8 (50%) of the patients with N2b OSCC.

### 3.5 Presence of metastasis in SN (SN+) in OSCC

Out of 85 patients, 84 had a histopathological report from the SN biopsies. Among the 84 patients, metastases (micro/macro or isolated tumor cells) were found in one or more SNs in 30/84 (35.7%), see (Table 1). Advanced OSCC (T3–T4 and/or N+) presented SN+ at a higher rate than early‐stage, 12/21 (57.1%) versus 18/63 (28.6%). The rate of occult contralateral nodal metastasis (SN+) in all stages of OSCC was 4/84 (4.8%): 2/63 (3.2%) in early‐stage OSCC and 2/21 (9.5%) in the advanced‐stage OSCC.

### 3.6 Positive SN (SN+) in relation to T‐stage

The number of SN+ increased with the T stage, regardless of the N‐stage, ranging from 20% to 75% (Table 4).

**TABLE 4:** Positive sentinel node (SN+) in relation to T and N stage.

SN+			
T‐stage	Total	N0	N+
ALL	30/84 (35.7%)	23/71 (32.3%)	7/13 (53.8%)
T1	8/39 (20.5%)	8/37 (21.6%)	2/2 (100%)
T2	12/30 (40%)	10/26 (38.5%)	1/4 (25%)
T3	7/11 (63.6%)	4/7 (57.1%)	2/4 (50%)
T4	3/4 (75%)	1/1 (100%)	2/3 (66.7%)

Despite few included T3 and T4 patients, it appeared as if SN+ was more frequent in T3–T4 N0 than T1‐T2 N0 (*p* = 0.1, 95% CI 0.917–16.650), a trend that became significant when comparing all T1–T2 versus T3–T4 (*p* = 0.0398, 95% CI 1.039–11.560) (Tables 4 and 5).

**TABLE 5:** Comparison of T‐stage and sentinel node positivity according to histopathology in oral cancer patients with (a) clinically N0 disease; (b) clinically N0 and N+ disease.

a. Patients with clinically N0 disease				
		SN‐ n (%)	SN+ n (%)	*p*‐value^a^
T‐stage				
T1+T2		45 (71.4%)	18 (28.6%)	0.1020
T3+T4		3 (37.5%)	5 (62.5%)	
Location of tumor				
Oral tongue and floor of the mouth		43 (69.4%)	19 (30.6%)	0.4581
The buccal and mandibular gingival tumors		5 (55.6%)	4 (44.4%)	

b. All patients (both clinically N0 and N+ disease)				
		SN‐ n (%)	SN+ n (%)	*p*‐value^a^
T‐stage				
T1+T2		48 (69.6%)	21 (30.4%)	0.0398
T3+T4		6 (40%)	9 (60%)	
Location of tumor				
Oral tongue and floor of the mouth		43 (60.6%)	28 (39.4%)	0.220
The buccal and mandibular gingival tumors		5 (28.5%)	8 (61.5%)	

^a^ Fisher's exact test.

### 3.7 Positive SN (SN+) in relation to N‐stage

Patients with N+ disease presented as SN+ at a higher rate than patients with N0 disease, 7/13 (53.8%) compared to 23/71 (32.4%). Patients with T1–T2 N+ demonstrated SN+ in 2/6 (33.3%) cases and patients with T3–T4 N+ had SN+ in 5/7 (71%) cases. Furthermore, while 2/5 (40%) with N1 disease presented as SN+ , 5/8 (62.5%) of the patients with N2b were SN+ (Tables 1 and 4).

### 3.8 Positive non‐sentinel node (non‐SN+) in the neck specimen

In 7/58 (12.1%) of all patients who underwent ND, we found an additional metastasis in a non‐SN in the histopathological report of the neck specimen. Additional positive non‐SNs were found in 3/40 (7.5%) of all patients with early‐stage OSCC who underwent ND, while non‐SN+ were found in 3/18 (16.7%) of patients with SN+ early stage, compared to 2/21 (9.5%) of all cases with advanced OSCC Among the patients with T3–T4 N0 disease, no non‐SNs were found (0/5), while 2/13 (15.4%) of patients with N+ OSCCs had non‐SN+. Importantly, all non‐SN+ were found in neck levels with previously known metastasis or SN+.

## 4 DISCUSSION

The risk of regional recurrence after elective and therapeutic neck dissection in patients with OSCC is relatively high, and the prognosis after recurrence is poor.^15^, ^25^ Thus, finding ways to reduce the rate of regional recurrence could substantially improve survival in these patients. While sentinel node biopsy is an established method to improve regional control in early‐stage T1–T2N0 OSCC, only a few studies have included a limited number of SNLB in patients with advanced T‐stage OSCC and when it comes to N+ OSCC the pattern of lymphatic drainage and the prevalence of SN+ has not been previously described. In this study, patients with advanced OSCC present contralateral lymphatic drainage at a higher frequency than patients with early‐stage OSCC. Furthermore, patients with advanced T3, T4, and N+ disease exhibited a higher rate of SN+ compared to patients with early‐stage disease. These results suggest that the use of SN‐technique increases the possibility to surgically remove all occult metastases even in advanced OSCC.

The increased risk of contralateral recurrence and occult contralateral metastasis after ipsilateral END is previously known and related to T‐stage, and some authors have even suggested bilateral END in patients with T3/T4 disease or N+.^9^, ^18^, ^25^, ^27^ Furthermore, when comparing neck failure in the contralateral side in early stage OSCC, patients treated with SLNB‐biopsy present lower numbers of failure compared to patients treated with END.^25^ In this study, we see a clear pattern of increased risk for contralateral lymphatic spread with higher T‐stage. Our results confirm the higher risk of contralateral spread in larger tumors. By combining traditional ipsilateral END/TND with SN‐technique for T3–T4 OSCC, possible lymph drainage to the contralateral side can be identified and included in the surgery by SNLB. SNLB on the contralateral side could result in a more accurate staging and could also in some cases spare the patients form the side effects caused by bilateral ND.

Also, we found differences in lymph drainage pattern between the different oral subsites, where tumors localized in the floor of the mouth (C040‐C049) have a higher risk of contralateral lymphatic drainage, followed by the tumors in the oral tongue (C020‐C023), while the risk seems lower for tumors in the buccal mucosa (C060) or mandibular gingiva (C031). In our study, there were no midline tumors, although few tumors were located close to the midline. Almost all small tumors that showed contralateral lymph drainage were located close to the midline or located in the floor of the mouth. However, there was one patient with a well‐lateralized T2 tumor and two patients with T4a tumors of the mandibular gingiva who also had contralateral SNs. This highlights the fact that contralateral lymph drainage can occur even in well‐lateralized tumors.^19^, ^24^, ^25^ Accordingly, our results are in line with previous studies on early‐stage OSCC where T1–T2 tumors close to the midline present contralateral lymph drainage at a higher rate than more lateralized tumors.^9^, ^28^, ^29^

It has previously been shown that neck failure accounts for a large number of recurrences in both early stage and more advanced OSCC. Recurrence occurs both in the ipsilateral neck, treated with END/TND with or without adjuvant RT/CRT, representing in‐field recurrences, as well as in the untreated contralateral neck.^15^, ^25^, ^27^ In patients with early stage OSCC treated with ipsilateral END, 69% presented in‐field recurrence while recurrences in the surgically untreated contralateral neck was found in 39%.^27^ Furthermore, patients with early stage OSCC who underwent END had a higher risk of developing contralateral regional recurrence than those who underwent SLNB, 3.8% versus 1.3%, and none of the patients where occult metastases were detected by SLNB developed contralateral neck recurrence.^25^ Consequently, the use of SNLB in advanced OSCC enables contralateral staging of the neck at the same time as the ipsilateral neck is addressed. Furthermore, SLNB may in some cases offer a less invasive alternative to bilateral ND for patients with tumors close to or crossing the midline, thus avoiding overtreatment of the contralateral neck by allowing accurate selection of those patients who may require treatment of the contralateral neck.^25^ In our study, none of the patients with N+ OSCC showed any presence of contralateral metastases after standard preoperative investigation including CT and/or MRI. After SPECT–CT, and confirmed with gamma probe and ICG during surgery, 43% (6/14) of all patients with N+ OSCC presented lymph drainage to the contralateral neck, compared to 30% (21/71) among patients with N0 disease. Interestingly, in six of the N+ patients diagnosed with lateralized tumors in buccal mucosa and mandibular gingiva, three presented lymph drainage to the contralateral side. The high proportion of contralateral SNs for patients with N+ and advanced T3–T4 OSCC, and the actual presence of positive SNs on the contralateral side (9.5%), shows clinical advantages of adding the SN technique to the traditional ND performed for OSCC with N+. Also, patients with advanced T‐stage and N0 will benefit from the SN‐technique since it allows including SNs from the contralateral neck. Another advantage of using the SN‐technique during the ipsilateral ND is the possibility to recheck the neck field at the end of the neck surgery, with gamma probe and ICG, to ensure that no SNs are left behind.

In previous studies performed on T1–T2 N0 OSCC, metastases were found in 9.2%–23% of the SNs,^19^, ^21^ which is in line with our results where 20% had SN+ in early OSCC. As expected, the rate of SNs + was higher in the advanced OSCC and an increasing rate was seen with higher T stage, and in N+ vs. N0 disease. It could be theorized that the node metastasis could interfere with the lymphatic tissue and its function and thereby obstruct the lymphatic drainage, subsequently blocking any signal uptake at SPECT CT and/or ICG‐imaging. However, in our study, all previously known metastases in patients with N+ OSCC, were confirmed by preoperative SPECT–CT and per‐operative gamma probe and ICG‐imaging. After performing SN‐assisted ND we found additional metastasis in SNs in 54% of the patients with N+ OSCC disease. In 60% of the cases the SN was found in another neck level than the known metastasis. The prevalence of SN+ additional to the known metastases confirms the importance of identifying and including all SNs in the surgery of the neck, both on the ipsilateral‐ and contralateral side. Therefore, adding the SN‐technique to advanced N+ OSCC gives us the opportunity to include all SNs in the neck surgery and possibly reduce the risk of recurrence for this group of patients. Furthermore, a more sensitive detection of contralateral metastasis improves staging and thus a more accurate post‐operative treatment (adjuvant RT/CRT) can be given.

The method of mapping the lymphatic drainage from the tumor differs between medical centers. In some centers, post‐injection imaging is done within 5–30 min from injection.^24^, ^30^ This technique can identify the velocity from the injection site to the first SN and subsequently to the other echelon nodes allowing the surgeon to identify and resect only the first SN and thus possibly lower the number of negative nodes harvested.^24^ Other institutions perform the imaging within 30–90 min from injection to include more of the tumors SNs and also to add more precise anatomical and spatial information on their localization.^31^, ^32^, ^33^ There is also evidence that it is important to not only harvest the SNs with the strongest signal, but also all nodes with signal, since metastases in several cases were found in SNs with weaker signals.^34^

In the literature, additional non‐SN+ are found in 15%–40% of the ND after positive SN biopsy in early‐stage OSCC,^16^, ^21^, ^24^, ^35^, ^36^ while in our study non‐SN+ were found in 7.5% in ND in patients with early‐stage disease and in 10% in the advanced OSCC cases. In a recent study by Panula et al., 33% of the SN+ were not found in the SN with the strongest signal, but rather detected in a SN with a weaker signal.^34^ This implies that including all SNs is important. At Karolinska University Hospital the SPECT–CT is performed earliest 1 h after the peritumoral injection of TC‐99 m labeled Nanocolloid/Tilmanocept, to ensure that all SNs are visualized, which may have an impact on the low‐rate of non‐SN+ in our material. ^26^ Including all tumor draining SNs may also have an impact on the rate of false negative SNs occurring as neck relapse, although studies with a larger patient cohort and longer follow up time is warranted. Furthermore, in previous data the rate of false negative SNs has been described as 15%, that is regional recurrences.^21^

In previous studies the majority additional non‐SN+ were found in the same neck level as the SN or adjacent neck level.^21^ In our data, based on prolonged time to SPECT–CT, no additional non‐SN+ were found in neck levels other than the previous known metastases or SN‐location, making us suggest that the neck dissection guided by SN technique should be customized to include the neck levels with identified metastases and SNs. Another future possibility is to guide the postoperative RT based on the SN‐mapping.^37^ More accurate surgery and radiation fields in the neck could potentially improve survival and in some cases diminish the patients acute and long‐term side effects. The low rate of non‐SN+ in early stage OSCC (7.8%) also raises the question if in the future it would be sufficient to excise only the positive SN basin without a therapeutic neck dissection for early‐stage OSCC, when using the proper technique? This remains to be studied further.

Both radiotracer and ICG dye, were injected submucosally around the tumor, in 4 or 5–6 sites, depending on the tumor size. In this study, we did not find it technically more difficult to inject patients with larger tumors. In this limited cohort, the number of non‐SN+ found in the ND of T3–T4 N0/ N+ patients were equal to those found in the ND from patients with T1–T2 N0, which could indicate that the submucosal peritumoral injections is applicable also for larger tumors.

SNLB is today incorporated in many national guidelines for early stage OSCC, even though it is not universally accepted. There are several limitations of the technique, that is false negative SN, risk for a second surgery in case of SN+, extra time and cost required to preoperative injection and imaging, time‐consuming histological analysis and surgical learning curve.^21^ Also, the accuracy to detect occult metastasis in floor of mouth tumors with SLNB is significantly lower,^16^ and there is limited data availed on the survival outcomes between SNLB and END.^22^ These limitations highlight the need for more research on SNLB in OSCC.

### 4.1 Limitations of the study

This retrospective study includes a limited number of patients, especially the group with advanced stage OSCC. Also, the short follow up time does not allow us to draw any conclusions regarding the outcome for regional recurrences/survival at this stage and longer follow up is needed.

Since the aim of this study was to highlight the possible advantages of adding the sentinel node technique to the surgical protocol even for the advanced stages of OSCC, we believe the results to still be of value.

## 5 CONCLUSIONS

Patients with advanced OSCC (T3, T4, N+) present a higher rate of contralateral lymphatic drainage and SNs+ compared to patients with early stage (T1, T2, N0) OSCC. The presented findings indicate that adding the SN‐technique to the neck surgery might be of clinical value in advanced OSCC since it enables a more accurate staging without performing contralateral ND, as well as increasing the possibility to surgically remove all SNs+. The precise localization of the SNs+ also suggests that a more individualized ND and RT/CRT approach might be possible even for the advanced OSCC, although further studies are warranted. Therefore, based on the findings of this study, we recommend using the SN technique in all stages of OSCC.

## ETHICS STATEMENT

All procedures performed in studies involving human participants were in accordance with the ethical standards of the institutional and/or national research committee and with the 1964 Helsinki declaration and its later amendments or comparable ethical standards. Informed consent was obtained from all individual participants included in the study. Ethics Committee Approvals: 2015/1650–31/2, 2019–03518 and 2021–0165.

## AUTHOR CONTRIBUTIONS

**Rusana Bark:** Conceptualization (equal); data curation (equal); formal analysis (equal); funding acquisition (equal); investigation (equal); validation (equal); visualization (equal); writing – review and editing (equal). **Aeneas Kolev:** Investigation (equal); writing – review and editing (equal). **Alexandra Elliot:** Investigation (equal); writing – review and editing (equal). **Krzysztof Piersiala:** Formal analysis (equal); software (equal); validation (equal); visualization (supporting); writing – review and editing (equal). **Anders Nasman:** Formal analysis (equal); methodology (equal); software (equal); writing – review and editing (supporting). **Per Gryback:** Methodology (equal); writing – review and editing (equal). **Susanna Kumlien Georén:** Writing – review and editing (equal). **Malin Wendt:** Writing – review and editing (equal). **Lars Olaf Cardell:** Funding acquisition (equal); resources (equal); writing – review and editing (equal). **Gregori Margolin:** Conceptualization (equal); investigation (equal); writing – review and editing (equal). **Linda Marklund:** Conceptualization (equal); data curation (equal); formal analysis (equal); funding acquisition (equal); investigation (equal); project administration (equal); resources (equal); supervision (equal); validation (equal); visualization (equal); writing – original draft (equal); writing – review and editing (equal).

## FUNDING INFORMATION

This research was funded by the ACTA OTO‐LARYNGOLOGICA FOUNDATION 221125; THE LARYNGFÖRBUDNDET FOUNDATION, grant number: K48‐19; THE SWEDISH CANCER FOUNDATION, grant number: 190287; THE CANCER REASEARCH FOUNDATION OF RADIUMHEMMET, grant number: 194062; and REGION STOCKHOLM (ALF), grant number: 954996.

## CONFLICT OF INTEREST STATEMENT

The authors declare no conflict of interest.

## Data Availability

The data presented in this study are available on request from the corresponding author. The data are not publicly available due to Swedish laws on personal confidential information.
